# Identification of large-scale genomic variation in cancer genomes using *in silico* reference models

**DOI:** 10.1093/nar/gkv828

**Published:** 2015-08-11

**Authors:** Sarah Killcoyne, Antonio del Sol

**Affiliations:** Luxembourg Centre for Systems Biomedicine (LCSB), University of Luxembourg, Campus Belval, 6, Avenue Swing, Belvaux L-4367, Luxembourg

## Abstract

Identifying large-scale structural variation in cancer genomes continues to be a challenge to researchers. Current methods rely on genome alignments based on a reference that can be a poor fit to highly variant and complex tumor genomes. To address this challenge we developed a method that uses available breakpoint information to generate models of structural variations. We use these models as references to align previously unmapped and discordant reads from a genome. By using these models to align unmapped reads, we show that our method can help to identify large-scale variations that have been previously missed.

## INTRODUCTION

Cancer genomes are diverse, and often differ considerably from their germlines. This means that the standard *reference-based* mechanisms used in genome alignment are not always suitable. These mechanisms rely on the assumption that the reference is highly similar to the sample genome. As the reference being used is not an accurate representation of the cancer genome(s), alternative strategies that can use references that represent large-scale structural variation are needed. This paper introduces one such method that uses prior information about known characteristics of cancer genomes to inform a search strategy, which allows for a more efficient mapping of reads against alternative references.

One of the problems with cancer genomes is that they exhibit a high degree of structural variation from the germline. Genomic structural variation is defined as alterations to the genome sequence such as duplication, copy number variation, inversion or translocation (1). While the size of small structural variants can range from anything over a single base pair to 1kb, large-scale variations can involve up to several million base pairs and result in chromosomal aberrations that can be seen at the microscopic level.

Prior to the advent of high-throughput sequencing (HTS) technologies, microscopic methods enabled the identification of cancer structural variation at the level of chromosomal aberrations. Large insertions, deletions or translocations could be identified in a karyotype using Giemsa staining, fluorescence-*in situ* hybridization (FISH), or spectral karyotyping (SKY), and associated with disease phenotypes. These large-scale chromosomal aberrations are rare in the population generally (due to developmental lethality in most cases) and are often associated with severe disease phenotypes. However, the number and complexity of these large variants can be high in tumor genomes (2–4).

In a number of cancers these microscopic levels of structural variation are clinically significant markers of tumor type and malignancy. Known variants can be used to stratify a patient's disease as in multiple myeloma with recurrent translocations between chromosomes 4, 11 and 14 (5), while others such as the Philadelphia chromosome in leukemia (chronic myelogenous and acute lymphoblastic) results in a clinically significant gene fusion BCR/ABL1 (6), which is used in targeted drug therapy (7). Additionally, it has been shown that mutational complexity, including chromosomal aberrations, increases over time contributing to an aberrant activation/repression of multiple genes and therefore potentially contributing to drug resistance or metastasis (8–10). This means that while many translocations (both intra- and inter-chromosomal) have been identified, an individual patient's tumor genome could display a complex mixture of structural variations which may not already be characterized.

**Figure 1.**
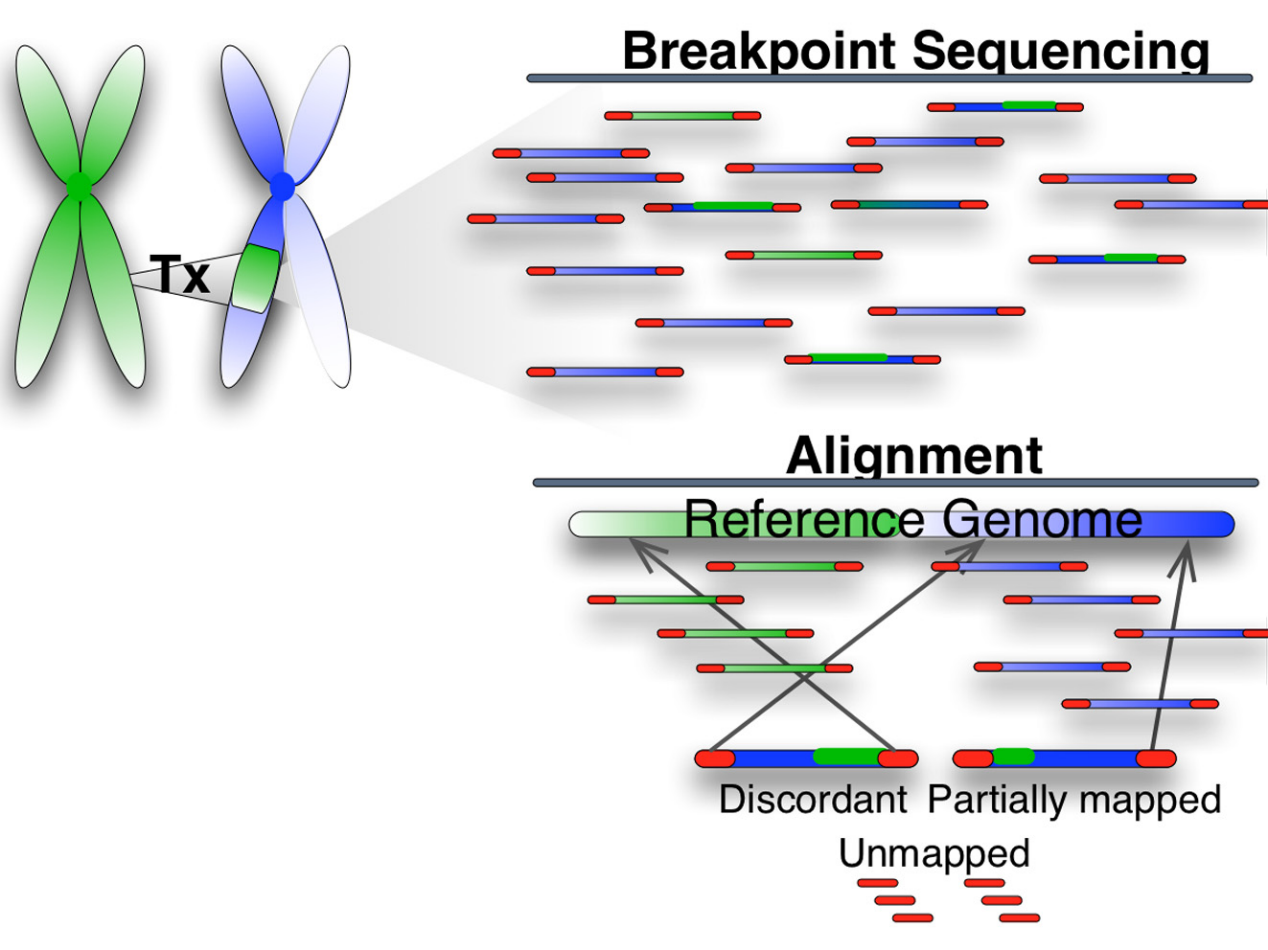
Intra- or Inter-chromosomal translocations result in read pairs that will not align with the expected insert size between the pair. Some of these may be aligned, but will lack information regarding the mapping quality, as that is dependent on the gap and location information. Many reads resulting from this sort of variant may also be unaligned. These issues are a direct result of the use of a reference sequence that does not reflect the structure of the sample sequence.

As sequencing has become a common method of identifying individual variants in both clinical and research labs, identifying large-scale variants from HTS data alone is increasingly important. There are still a number of issues in identification of large-scale variants in short-read sequence data. The first issue is due to the small size of reads relative to the variation. The current generation of HTS technologies were developed to enable the rapid sequencing of entire genomes through the parallel sequencing of overlapping short-reads (11). In pair-ended HTS short segments are sequenced (e.g. 35–250 bp for Illumina) from two ends of a fragment of known length (e.g. 200–800 bp for Illumina). When aligning these reads to a reference the insert size between each read pair allows the alignment algorithm to indicate that a read-pair is correctly aligned to a specific location (12). However, these locations are dependent on finding a good alignment to the provided reference, and in the case of a cancer genome with large-scale structural variation such a reference will be a poor fit (see Figure 1).

In large-scale variations a breakpoint and recombination occurs at a potentially fragile location on the chromosome, altering the sequence. A read-pair generated from this genome can span the breakpoint (if it happened to fall within the gap of the read-pair), or result in a ‘split’ read where the beginning and end of the read align to different locations. However, due to computational limitations inherent in sequence alignment many reads that could identify these breaks may not be mapped to the reference (13,14).

Complicating the already difficult task of identifying large-scale structural variants in tumor samples is the high degree of genomic heterogeneity present. Samples taken from a solid tumor can include multiple sub-clonal cellular populations that do not share the same variants (15). The result in a sequencing sample is a low frequency of reads supporting a given variation, and in large structural variants some or all may also be unmapped and therefore unavailable for identification.

**Figure 2.**
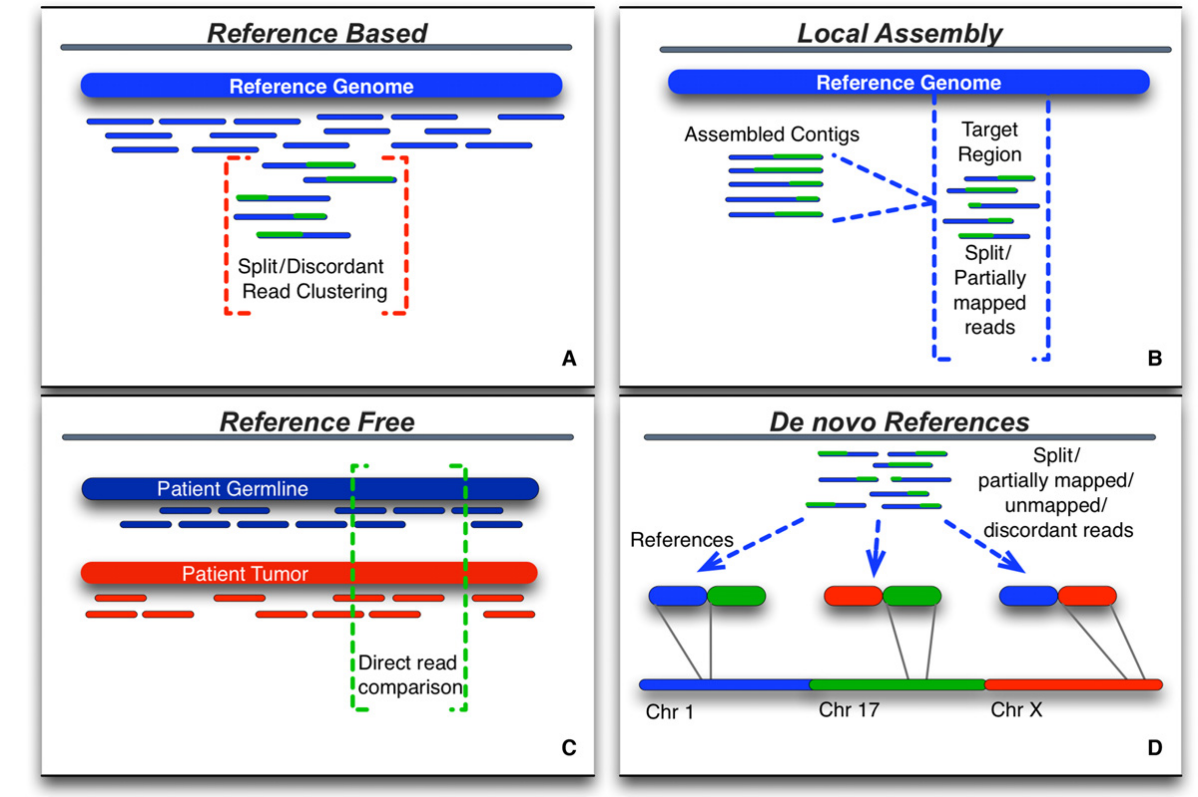
Structural variation detection methods have generally used the *reference based* (**A**) approach where reads are first aligned to the reference, then clustered using a variety of measures to identify SVs. Most of these methods suffer from the assumption that the alignment of the read that is reported is the correct one, due to computational limitations of the alignment algorithms. Alternative methods proposed include performing a local realignment (**B**) of misaligned reads after building longer contigs of the putative breakpoint regions (e.g. *Local Assembly*), and directly comparing the reads of two or more genomes (**C**), in this case tumor/normal, to identify mutations ranging from single nucleotide to inter-chromosomal translocations (e.g. *Reference Free*). Our method (**D**) aims to identify breakpoint regions by generating multiple small references which model potential breakpoints (e.g. *De novo References*), complementing the existing methods.

These difficulties have resulted in a variety of methods being developed which use short-read sequencing data to identify structural variants. The most commonly used methods are *reference based* (see Figure 2A) where variant analysis relies on the initial alignment of sequence reads to the reference genome. When using the aligned reads the existence and position of a breakpoint, and the resulting structural variation, is inferred through clustering or windowing strategies (16). The ‘discordant’ reads (e.g. mapping to different chromosomes or with incorrect orientation) are used to identify a possible breakpoint through clustering the reads as in BreakDancer (17) or Pindel (18). While PRISM (19), DELLY (20) and SoftSearch (21) cluster ‘split-reads’ where one of the pair has mapped unambiguously to the reference genome or the CIGAR value has significant numbers of soft-clipped bases (e.g. partially aligned reads). As these methods often limit the size of the variants they can detect consensus approaches such as SVMerge (22) are often used to increase detection across all types.

While the *reference based* approach is widely used, it suffers from a number of limitations inherent to current alignment algorithms, and these methods are therefore unable to identify many structural variants in highly heterogeneous samples. These limitations include the current short length of read-pairs along with the highly repetitive sequence of the human genome. This makes it highly likely for a read pair to align to multiple locations (23,24) across the genome. Alignment algorithms most often use the ‘best mapping’ approach to reporting read alignment, where the alignment resulting in the fewest mismatches is reported or when all are equally good matches one is randomly selected (25). This is due both to algorithmic constraints, as allowing mismatches increases the number of possible alignments, and to simplify downstream computation as the reads with the highest quality scores are used in variant detection (26). *Reference based* alignment algorithms (e.g. BWA (27), Bowtie (28), SOAP2 (29)) cannot practically do an exhaustive search in the case of reads which may have multiple alignments (as can happen with high rates of variation or with too many mismatches), and where alignments are found will typically only report one of many possible alignments. This is a fundamental limitation to methods that rely on reference alignment, especially when considering tumor genomes. Thus alternate methods that rely less on the reference genome are now being developed.

In the last two years, two alternative approaches have been published which do not rely directly on the standard reference genome alignment. The first approach is best described as a *Local Assembly* (see Figure 2B) method, developed by Abo et.al. (30). This method reassembles misaligned reads within a target specific region into contigs using an overlapping kmer seed from the sequence reads and target region. These contigs are then realigned within the target regions and classified into specific variant types (e.g. inversions, indels, translocations). Locally assembling contigs from regions that have high rates of misalignment overcomes one of the major issues inherent to short-read sequencing, namely that the read lengths are too short to uniquely align when the genome has been structurally altered. While this approach cannot currently scale genome-wide, as it effectively involves *de novo* assembly, it is ideal for resequencing experiments or targeted identification in whole genome or exome data.

The second alternative is the *reference free* (see Figure 2C) method, which takes a completely different approach and avoids the reference entirely by directly analyzing the reads without first aligning them. In this case there is no positional information known, and here the methods vary widely in their implementation. Hormozdiari et.al. (31) assumes that structural variants can be detected with higher accuracy by using multiple related genomes. In this case while a reference genome is used as an intermediary in the analysis, the authors assume that the true variants are discoverable by simultaneously comparing patient genomes directly. They show this clearly with small structural variants (<1 kb) in several genomes from the YRI population in the 1000 Genomes data (32) and a family trio, though it is less clear how well this may work in complex tumor samples. A more recent *reference free* approach called SMUFIN (33) directly compares reads without alignment and was developed specifically for the tumor/normal pairs of genomes. Here it is expected that reads will be highly similar and mutations can be found by grouping reads into a tree structure that branches where mutations are found. Breakpoints can be identified in the branches of the read tree, and local alignment performed. Both *reference free* approaches identify structural variation with greater accuracy than the primary *reference based* approaches.

The analysis for SMUFIN also showed that there might be significantly more complex large-scale structural variation in tumor sequences than has been previously reported. This is due in large part to the fact that tumor genomes can include highly complex low frequency variations and the reference genome that alignment algorithms rely on cannot model these in mixed samples. The methods that rely on the *reference based* alignment (e.g. BreakDancer, SoftSearch, etc.) are limited by the aligners and, as is shown by both the *local* *assembly* and *reference free* methods, alternative approaches are necessary to overcome the alignment issues.

Here we propose a third alternative for identifying structurally variant regions, which can complement the existing methods in complex tumor samples: *de novo* generation of multiple references. Our *de novo* method (see Figure 2D) generates a large number of new references that model potential structural variations. We use a tuned optimization strategy based on prior information from karyotypes across many different cancers to select suitable references. Standard alignment tools are then used to align previously unmapped and discordant read-pairs to the new references, and the resulting alignments are scored. Using high-performance cluster computing this process can be repeated hundreds of times to select likely breakpoint recombination regions.

In the *Material and Methods* section, we describe our strategy starting with the generation of *de novo* references followed by identification of regions that may include structural variations. In the following *Results* section, we show that our identification method can find structural variants in simulated data and then we apply it in several patients from The Cancer Genome Atlas (TCGA) (34). Finally the *Discussion* section discusses the need for alternative strategies to identify structural variations in tumor samples and specifically the advantages and limitations of our *de novo* method.

## MATERIAL AND METHODS

**Figure 3.**
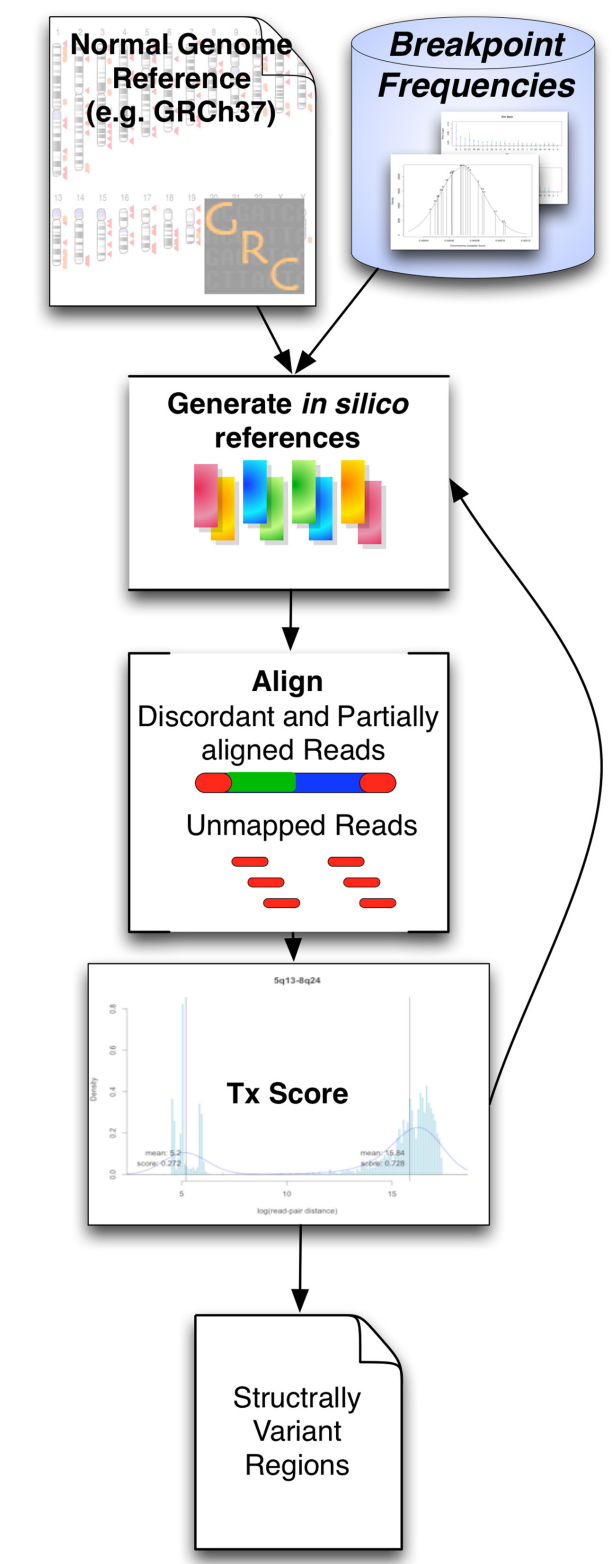
The general workflow of this method takes prior knowledge of the likelihood of an aberration at points in the chromosome to generate a new *in silico* reference. This is then used to align the discordant and unmapped reads from a previously aligned sample, scored and clustered to identify the best scoring regions.

The strategy we have used to enable more accurate alignment of cancer genomes from short reads is to limit the search space in which reads can align by altering the reference (see Figure 3), and therefore decreasing the read distance between potential split read-pairs.

In order to limit the search space while using standard alignment tools, the reference is replaced by a series of *in silico* reference sequences that model chromosomal recombination regions. The generation of *in silico* references requires a pre-populated database of breakpoint frequencies, including chromosomal locations generated from available breakpoint data, and are used to align only those reads in a sample that were previously partially or fully unmapped or discordant.

### *In silico* model generation

Instead of aligning against a single reference, our method aligns against hundreds of smaller references. These smaller references model potential structural variations seen in cancer originating from fragile regions in the genome. The set of new references contain sequences from two different genomic regions thus simulating the result of a recombination event. These models are generated using prior knowledge of breakpoint frequencies in cancer based on karyotype data (e.g. breakpoints at cytogenetic bands). These frequencies were obtained from analysis of public karyotype data sets including patient karyotypes and cell lines:

- ***Patient karyotypes***. 99 764 across many different (poorly curated) cancer types were analyzed from the Mitelman CGAP database (35) and 325 from NCI and NCBI's SKY/M-FISH and CGH Database (http://www.ncbi.nlm.nih.gov/sky/skyweb.cgi). The majority of these were blood cancers (e.g. leukemia, lymphoma and myeloma).
- ***Cell line karyotypes***. 84 were analyzed from the University of Cambridge CGP SKY/FISH of Epithelial Cell Lines (http://www.path.cam.ac.uk/∼pawefish/) and 67 from the NCI Fredrick National Laboratory NCI60 Cell Line Drug Discovery Panel (http://home.ncifcrf.gov/CCR/60SKY/new/demo1.asp). These were curated simply based on the tissue involved (e.g. ‘heart’ or ‘thymus’).

There are 320 major cytogenetic bands within the human genome (36), all of these are found to be involved in at least one breakpoint reported within the available data sets. A pairwise combination of each of the bands to create simulated references results in *C*_320,2_(51 040) possible combinations. While this would not seem to be too many combinations to test, and ideally testing against all of them would offer the most comprehensive view, there are computational limitations. First is disk space: the index for all simulated reference combinations requires 2.5 TB of hard disk space, and the subsequent alignment BAM files for a small set of reads (1.9 million) from a single genome would require more than 30 TB on disk. The second limitation is the alignment step itself. Aligning a small number of reads against many smaller references is an ideal situation for parallelization, however each single alignment (e.g. bwa mem –a –t 12) plus analysis computation still required 65 min on a single node in our local cluster. All 51 040 pairwise regions would require 840 000 compute hours (or 96 years) in order to align and analyze. Therefore even with access to a HPC cluster and a high degree of parallelization, this is a computationally intensive method.

Instead of using all possible *in silico* references we use an informed search strategy. This informed approach is required to select regions that should be tested for breakpoints. As each breakpoint is not equally likely based on the karyotypes described, and to further decrease our search space and computational load, the frequencies calculated from karyotype data are used to generate a set of several hundred simulated references. This informed approach is outlined in the *Optimisation of Reference Selection* section below.

### Structural variant detection

The generated references now act as model regions for possible large-scale structural variation. Limiting the search space by creating smaller references also allows us to increase the number of possible alignments by including previously unmapped reads. A filtered set of reads from a patient sample that includes only those reads that were already aligned to different chromosomes (‘discordant’) or where one or both reads were unmapped are then aligned to these smaller references in parallel. This enables the method to rapidly compare multiple possible recombination regions. As we have limited the search space by using a smaller reference we are able to relax the search criteria to allow for more exhaustive searching and greater mismatches.

**Figure 4.**
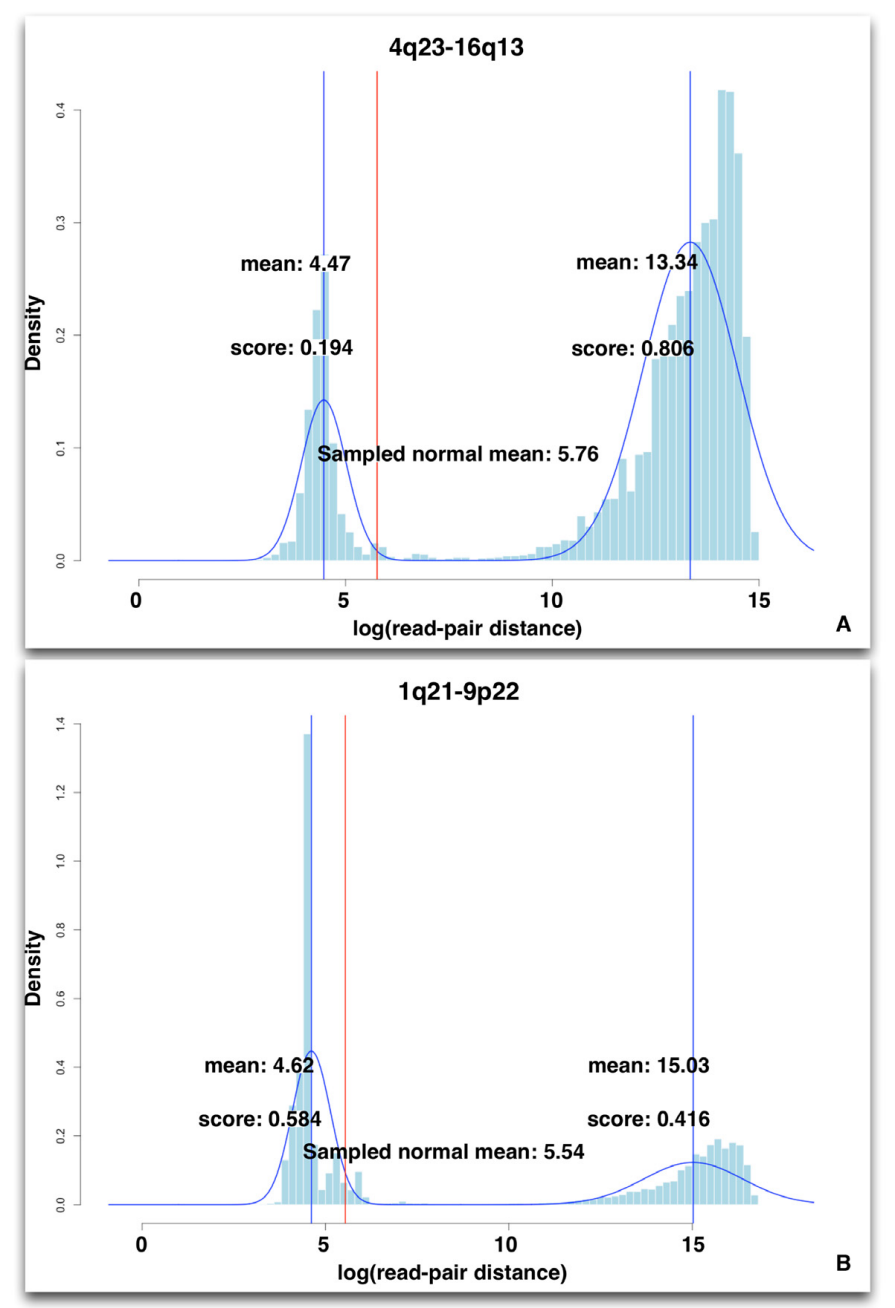
These plots show representative distributions of logged read-pair insert sizes from aligned reads against two different *in silico* references. Both aligned references show a bimodal distribution with two clear centers, however (**A**) shows an alignment to a reference that may be representative of a SV in the sample, while (**B**) is representative of an alignment that shows only noise based on the EMr values for the second distribution in each plot. In both (**A**) and (**B**) the first distributions (on the left of each plot) are from read-pairs that align with an insert size <2 s.d. from the mean, while the second distributions (on the right of each plot) are from read-pairs that align with an insert size >4 s.d. from the mean. In (**A**) the aligned reads in the second distribution show a very clear signal with a large number of supporting reads, suggesting there is a SV within this region. In (**B**) the second distribution is poorly defined. The reads in this region are less likely to indicate a SV as the second distribution does not exceed the noise from the first.

In each model region the aligned reads are filtered to limit the inclusion of poor quality data. As these reads were previously unmapped, we filter out reads from the alignments that are below the mean summed Phred quality score identified from the original BAM. Additionally, any alignments where 50% or less of the read have matched according to the CIGAR (see SAM format) value are discarded. Each model region is then evaluated by analyzing the distribution of read-pair insert sizes in each new alignment. A bimodal distribution of the logged insert size between aligned read-pairs is observed across the *in silico* reference alignments. In all model regions, the distributions are consistently bimodal and non-symmetric, and each peak is reflective of these two different possible alignments (see Figure 4). We find these sub-distributions using the Expectation-Maximization (EM) algorithm (R package ‘mclust’ (37)). The first distribution is characterized by reads with a small insert size (<2 s.d. of the mean insert size) and which are poorly mapped, having a map quality score <30. This indicated that the first distribution is basically noise, and arises due to alignments with a high likelihood of error. The second distribution includes reads that consistently align with an insert size that was >4 s.d. from the mean insert size, as used in (16). Due to insert size there is no map quality score, but we can overcome this by including only those alignments with higher CIGAR and Phred values.

The individual *in silico* reference score is calculated in two parts. The first is based on the mixture model parameters for the second distribution as calculated by EM:

\begin{equation*} EMr = \frac{{\sum\nolimits_{n = 1}^N {P(n|z)} }}{N} \end{equation*}

Where *P* (*n*|*z*) is the conditional probability of the *n*th read belonging to each of the two distributions identified. The *EMr* reflects the proportion of reads that are found to have a higher mate pair distance, and is derived by finding the probability of the *n*th read belonging to the second distribution, then iterating over the set of *N* where *N* is the total number of reads aligned to this reference. The resulting value is a ratio based on the number of distributions found and the sum of the EMr for each is 1. As the first distribution describes ‘noise’ in the alignments we can use it to find a cutoff value for further analysis of the alignments described by the given *in silico* reference. All models where the second distribution have an *EMr* below the cutoff can therefore be discarded.

The second part of the score is based on a sliding-window clustering approach to identify breakpoint locations based on alignment positions. Discordantly aligned reads from the second distribution are clustered by position if the read pairs also span both chromosomes represented by the simulated reference. This provides an estimation of windowed depth-of-coverage as discussed in (16) for a translocation breakpoint. However, this is not meant to provide a direct analysis of the breakpoint location, instead this provides a necessary adjustment for the EM score in the second distribution above.

\begin{equation*} Tx = EMr + \frac{{W_{max} }}{{N_b }} \end{equation*}

Where *W_max_* is the cluster with the highest total count of reads from the second distribution, and *N_b_* is the total number of reads within the second distribution.

### Simulated data sets

In order to estimate sensitivity and specificity for the *Tx* scores and subsequent structural variant calling, we generated reads using the ART (38) read simulator for Illumina in 20 sets of randomly selected pairs of chromosomes and cytogenetic bands. Each set included a randomly selected inter-chromosomal translocation based on position and sequence information from genome assembly GRCh37 (see Supplementary Table S1). The only limitation placed on the simulated breakpoints was that they did not fall directly on a cytogenetic band boundary and that they were in a region that could be aligned (e.g. avoiding poorly sequenced or highly repetitive regions such as most centromeres and telomeres). The analysis of these data is discussed in the *Results* section.

### Optimization of reference selection

As noted above, there are tens of thousands of pairwise combinations possible for just the major cytogenetic bands. Using probabilities to generate the most likely combinations will result in identifying reads that belong to well known breakpoints, while missing those that are less well characterized or are unreported in the literature. This means that in order to optimize the selection of simulated references, and avoid bias toward the most commonly known breakpoints (e.g. centromeres are the most reported breakpoints in the microscopic methods, or the Philadelphia chromosome in leukemia), a selection algorithm is introduced to generate populations of breakpoints. These populations are generated as individuals with full chromosomal complements and aberrant chromosomes.

**Figure 5.**
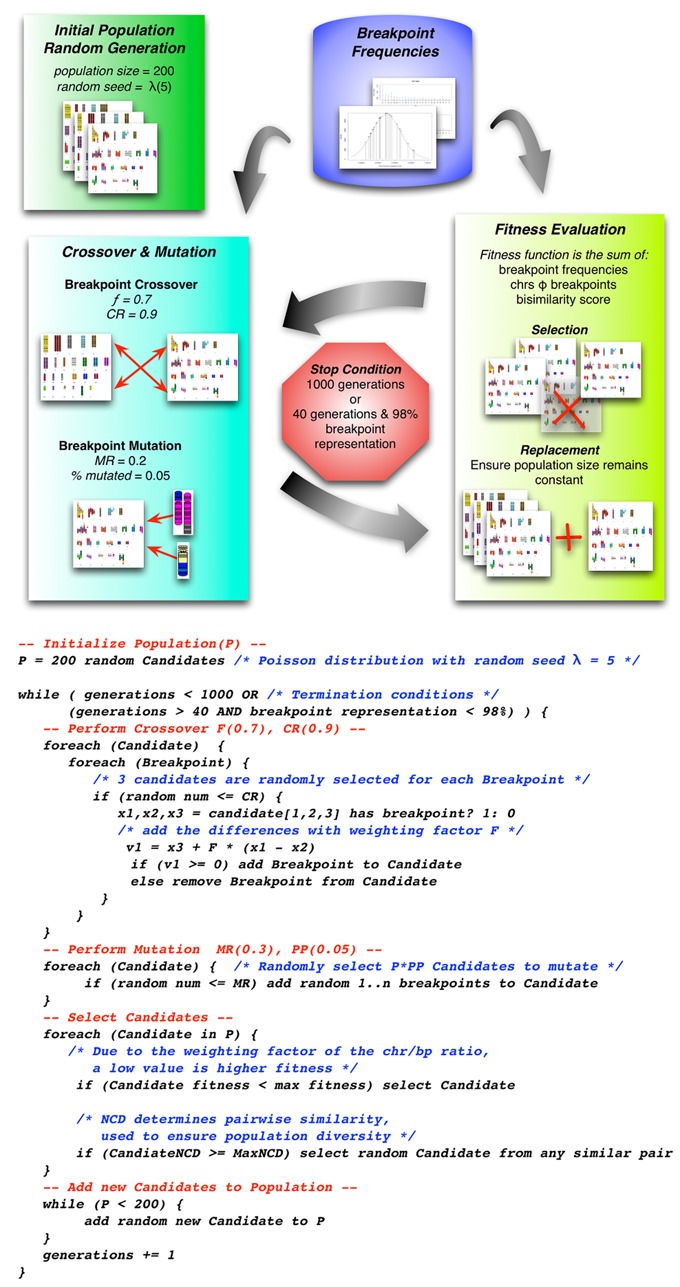
The selection algorithm is an implementation of differential evolution as this variant of genetic algorithms provide multiple solutions across the search space. The process of DE can be summarized as: (**i**) generate initial population (**ii**) cross each breakpoint pair by exchanging partners given a crossover constant (*CR*) (**iii**) mutate each breakpoint pair given a mutation constant (*F*) (**iv**) evaluate the individual fitness (**v**) evaluate the population diversity. When either the population diversity reaches a reasonable optimum or a certain number of generations have been run the selection algorithm stops. The parameters *CR*, *F* and maximum generations were all selected to optimize the diversity of the end population. Each of these constants can also have a large impact on the computational time it takes to generate a population.

This selection uses a type of genetic algorithm known as differential evolution (DE) (39) with an optimization function for the entire population being iterated over, instead of a single solution. This function combines the fitness of all individual references, and a measure of the diversity (see Supplementary Methods) of the DE population (see Figure 5). The diversity score ensures that cytogenetic bands with a smaller probability of being involved in a recombination event may be represented, enables the testing of chromosomal regions that may otherwise be underrepresented due to a bias in the frequency data (e.g. missing data for disease-specific aberrations), and avoids over-testing breakpoints that may be overrepresented in the knowledgebase (e.g. centromeres, Philadelphia chromosome, etc.).

The output of the selection algorithm is a population of pairs of chromosomal locations to be used in generating FASTA files. Each of these represents the sequence of the selected recombination. For example t(16;8)(q13;q24), is defined as starting with 16q13 (56700001- 57400000) and ending with 8q24 (117700001- 46364022) creating a recombination point at 700 kb.

## RESULTS

We performed a test on simulated data to validate the method and identify suitable parameters for cancer variant selection. Then we applied the parameters we learned from the simulated validation tests to tumor/germline data sets from TCGA directly. For the patient data sets we compare our method to BreakDancer, as it continues to be the most commonly used tool for large-scale variant detection in tumors.

### Simulated inter-chromosomal breakpoints

**Figure 6.**
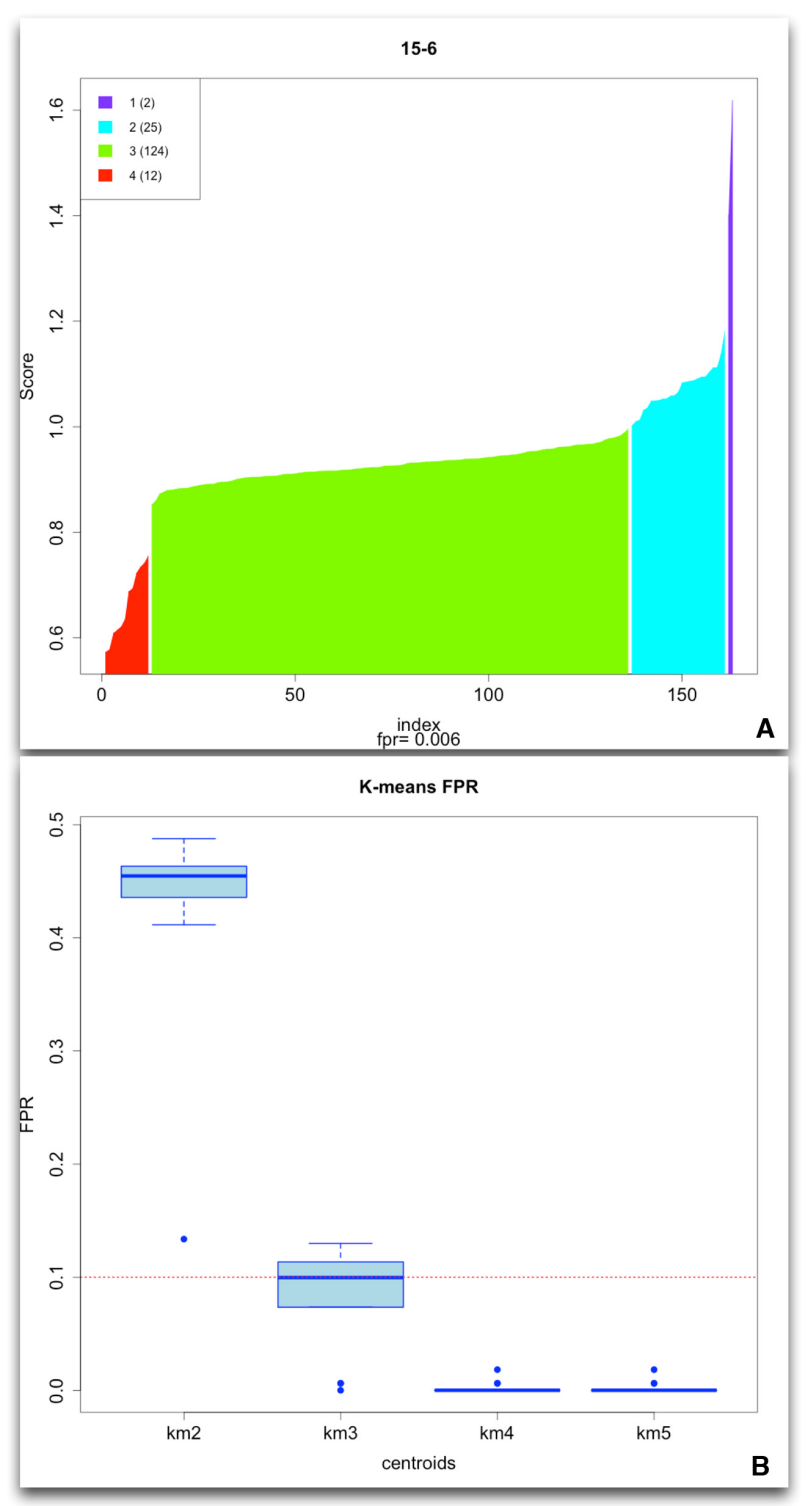
The *Tx* scores reported by our method (**A**) showed a one-tailed distribution. Using k-means we can identify the set of regions with the high *Tx* scores as shown in the purple cluster. This cluster consistently included the simulated breakpoint in all data sets across multiple coverage tests. From the 20 simulated data sets we determined that a selection based on 4 clusters (**B**) (seeding the centroids with quartiles), resulted in a FPR between 5% and 10%.

To validate that our method could identify inter-chromosomal breakpoints with a reasonable degree of accuracy we used the simulated data described in the *Methods* section. In the fully simulated set of 20 chromosome pairs we wanted to validate that the inserted breakpoint does result in a high *Tx* score when compared to a set of randomly selected references representing other potential breakpoint alignments. We applied k-means clustering to identify the set of regions with high *Tx* values. In order to keep the false positive rate (FPR) consistently below 10% in subsequent analyses we perform clustering using 4 centroids (see Figure 6).

As our method does not try to select a single unique alignment for each read, we expect to find a higher number of possible structural variations and therefore select a stricter cutoff to avoid increasing the likely errors. Additionally, the weighting value of the clusters in the *Tx* score $\left( {{\rm e}{\rm .g}\;\frac{{W_{max} }}{{N_b }}} \right)$ becomes more important in lower coverage or poorer quality simulations. This provides a useful parameter for investigation of structural variants, which are found in smaller sub-populations within the tumor.

In each of the 20 simulated data sets clustering the *Tx* scores resulted in identification of the known breakpoint in the top cluster of each set. It also enabled calculation of FPR values based on the clusters. These values were used in subsequent analysis of the TCGA patient data.

### Analysis: detection of large-scale variants in TCGA patients

We applied our method to the analysis of nine different matched tumor/germline genomes from TCGA across seven different cancer types (see Supplementary Table S2). Each set of unmapped and discordant reads from the genomes was compared against the same set of 278 regions selected by the optimization algorithm (see Supplementary Table S3). Based on the FPR rate calculated in the simulated data set above, we used a result selection from k-means clustering and took only the top cluster for analysis. We then filtered the germline hits from the tumor list in order to compare with BreakDancer. In each patient we identified regions that score highly for breakpoint inclusion. By using the germline samples to filter the results of the tumor samples we were able to remove regions that appeared to have significant unspecific read alignments as they were often found in both tumor and normal tissue samples.

Two patients had been previously analyzed as part of a large cohort study for TCGA: the glioblastoma (GBM) patient (40) and one of the colon/rectal (COAD (2)) patients (41). In these two patients we found no regions that were highly scored over their germline, which was consistent with earlier analyses. Our analysis of the GBM patient found no somatic structural variation, which was consistent with the cohort analysis where no structural variants were found in this patient. Our analysis of the COAD patient also found no structural variations. This was also consistent with the original COAD analysis as this patient was not found to have any structural or copy number variations.

It is also worth noting that in the ovarian patient (OV) samples our method identified two bands that are found as part of multiple regions (9q13, 4q13), which may suggest complex rearrangements. The 9q13 band is a known fragile site that is commonly involved in pericentric inversions in the germline linked with ovarian cancer (42), while 4q13 has been found to have a high rate of copy number variation in BRCA1 associated ovarian cancers (43).

### *Reference based* comparison

We compared these results to the BreakDancer analysis of tumor/germline pairs (see Table 1). Each translocation identified by BreakDancer was mapped back to the corresponding chromosomal region. Several findings are important here:

- ***Commonly identified regions*.** Structural variations that are found commonly across cancer types are highly likely to be due to biases in the detection method rather than a set of rearrangements that are common across cancer types. Both BreakDancer and our *de novo* method are going to find these due to the reporting of aligned reads. However, within the highest scoring regions, BreakDancer tends to find breakpoints in the same regions across multiple patients and cancers. Across all 32 regions, 26 were identified in more than one patient. For instance in the top regions with the highest scoring breakpoints, translocations in the 1p11–17p11 region were found in 6 of the 9 patients, while breakpoints in 1p11–11p11 are found in 4. In fact in the COAD-2 patient, where our method found no difference between tumor/germline, BreakDancer found only one region (3q27–6q15) where both bands involved were not identified in any other patient. Our method will also find regions that are common across cancers if the same set of regions are tested, however it is less likely with only 7 of the 29 regions being found in more than 1 patient, and the most common one (3q12–8p11) found in 4.
- ***Centromeres overrepresented*.** Across all 9 patients and cancers, centromeric regions are overrepresented in the top scoring breakpoints found by BreakDancer with 27 of the 32 regions including at least one centromere. Furthermore, in 7 of the patients all of the top 20 identified regions include at least one centromere. As centromeres (e.g. q11 and p11) make up only 15% of the major cytogenetic bands we would expect to see only 10 regions in 32 include a centromere in an unbiased sampling. As with the commonly identified regions, this is unlikely to be due to a common cancer event. Centromeres are poorly characterized across the chromosomes due to their highly repetitive sequences (25). This makes it more likely that reads aligning within a centromere will have correct alignments elsewhere in the genome. In fact the region shared across most patients (e.g. 1p11, found in 40% of the top regions) is poorly sequenced with 80% of the bases lacking a known assembly. Comparatively, our *de novo* method finds only 8 centromeres in our top 29 regions.
- ***Inaccurate alignments*.** Of the regions identified by BreakDancer with the highest scoring translocations in BRCA (breast cancer) two include alignments to the Y chromosome, however associated clinical data lists this patient as female. While this is not impossible, it would suggest issues with the alignment. As our method is not relying on a single reported alignment we did not find a similar inaccuracy.

**Table 1.** Shows the regions in which BreakDancer and our *de novo reference* method detect large scale structural variations

BRCA (1)	BRCA (2)	COAD (1)	COAD (2)	GBM	KIRC	LUAD	OV	LAML-14
*Reference based: BreakDancer*								
**1p11–19q11**	1q12-Yq11	1q12-Yq11	1q12–21p11	**1p11–17p11**	**1p11–17p11**	**1p11–19q11**	1p22–17q12	**1p11–17p11**
	1q21-Yq11	1q43–10p11	1p34–6p22		1q21–4p11	**1p11–6p11**	1p22–9q22	**1p11–11p11**
	**1p11–17p11**	1q21-Yq11	1q21–16p11		1q12–21p11		**1p11–11p11**	
	1q21–4p11	1q21–4p11	**1p11–17p11**		**1p11–11p11**		1q21–16p11	
	1q21–16p11	1q21–21p11	3q27–6q15				**1p11–17p11**	
			**1p11–11p11**				1p34–6p22	
*De novo references*								
Xq21–9q13	17q23–4q13	3q12–8p11			3q12–8p11	11p15–12q11	3q12–8p11	4q23–16q13
	4q13–2q14	11p15–12q11				6q22–11q21	10p14–9q13	3q12–8p11
		10p14–9q13				Xq21–9q13	17q23–4q13	2q13–14p13
		7p11–13q14				6q15–10p13	22q13–9q13	3q27–6q15
		Xq21–9q13				21q22–14q22	5q32–9q13	17p11–13p12
						10p14–9q13	4q13–2q14	5q32–9q13
						9p22–14q21		
						5q33–9p23		

The table shows representative results from nine different patient samples, all from TCGA. Regions in bold are those that occur in more than one patient, and regions in red under the breast cancer sample are those that are potentially erroneous (as they include alignments in the Y chromosome, where the clinical information list this patient as female). BreakDancer results show an overrepresentation of structural variations in centromeric regions in their top scoring translocations. The bolded regions all include the centromere 1p11, which is poorly sequenced with 80% of the bases missing in the current assembly. The *de novo references* method also results in a few shared regions and centromeric regions (e.g. 3q12–9p11 shared in 2 patients), however, it also finds more regions that include structural variation in the gene rich regions of the genome.

The highly duplicative nature of the regions that are the most commonly found in translocations by BreakDancer suggests that only very common breakpoints are found, and that there are issues with alignment. Alignment algorithms typically report only a single ‘best’ alignment leaving BreakDancer, and other *reference based* methods, with limited information on which to make identifications in complex samples. This is not to claim that the identifications are necessarily incorrect. Centromeres are likely to be involved in various types of large-scale structural variations due to microtubule defects (44), and it is not unlikely that certain regions are ‘hot spots’ for breakage and recombination. However, as reported by both SMufin and BreaKmer, *reference based* methods (and BreakDancer specifically) are missing large numbers of structural variants due to their reliance on the alignment algorithm.

As our method uses multiple *de novo* references to model potential breakpoints prior to alignment, we find regions with variation that *reference based* methods such as BreakDancer cannot. Thus while *reference based* methods provide a good initial estimate for variations, concurrent use of *reference free* or *de novo reference* *based* methods can provide a more complete view of the variation present in the tumor.

One of the difficulties present in the analysis of structural variation in cancer, is that in the absence of a directly gene related product (e.g. gene fusion) the effects may be subtle. For instance, if the break and recombination of a translocated segment occurs at intergenic regions the translocation may only affect the regulation or transcriptional enhancement of a gene rather than entirely inactivating the gene or increasing gene expression. Alternatively, as tumor genomes are often made of up highly heterogeneous cellular populations it is also likely that the effect of a fusion occurring in a sub-clonal population is at levels below our ability to detect in gene expression studies. It is therefore worth noting the regions identified by our *de novo* method may not result in fusion genes such as BCR/ABL but in altered regulation, or no detectable change.

In the second breast cancer patient (BRCA (2)) band 4q13 was represented in both of the top scoring regions, suggesting that alignments in that band were the main driver for the high scores. We generated an additional 10 regions that included 4q13 and added them to the pool of regions then performed the clustering again. All regions that included 4q13 were highly scored in the tumor sample, but not the germline. This is important to note for a few reasons. First, 4q13 is one of the regions known to integrate viral DNA from human papillomavirus (45) suggesting that there may be fragile sites for other types of structural rearrangement (and making it an important region for cervical and ovarian cancers as well). Secondly, several genes important to the development or aggressiveness of breast cancer including EREG, which is involved in ER/HER2 status, are located within this band. Finally, while the top match, 17q23–4q13, has not been reported as a structural variation previously both bands have been identified as showing significant copy number gains in aggressive breast cancers (46).

## DISCUSSION

The method introduced in this paper has a number of advantages and limitations. The main advantage is that our method is able to find structural variations that most commonly used tools would be unable to find. The reason these methods are unable to find these structural variations is that they lack the information required to identify them, as an exhaustive search during alignment is computationally inhibitive.

The limitations of our method are due to the fact that the increase in the number of references increases both the noise and search space. Aligning to multiple references results in multiple alignments reported for a single read, increasing the potential for noise. While this is an issue with the method, it is also a necessary condition for the identification of structural variations in complex samples from a tumor. By applying EM and a clustering parameter the regions with the highest likelihood of variation can be selected with a reasonable FPR. Secondly, this is a computationally heavy approach in that there are more possible combinations than can be reasonably tested. However, using prior knowledge and the search optimization algorithm we can limit the search space for each genome tested. This means currently we may miss regions that have structural variation as we do not search all possible regions instead using HPC tools and optimizations to decrease the overall time and computational load. By limiting our search both to the set of unmapped and discordant reads, and using a set of model references based on prior information, we are able to evaluate many possible regions. Ultimately this increases the ability to identify low-frequency aberrations likely to be present in heterogeneous tumor samples.

The identification of large-scale structural variation in cancer genomes continues to be difficult. Most of our current strategies have relied on alignment to a reference that is built on a ‘normal’ genome, assuming that the sample genome will align well enough for analysis. Unfortunately, due to the potential complexity of large-scale variations, the heterogeneity of a tumor sample itself, and the limitations of short-read sequencing, use of the standard reference can result in poor alignment for large-scale variant regions.

Alternative strategies that rely less on a reference genome, or skip alignment entirely, have provided evidence that the current *reference-based* methods cannot provide a complete view of the range and complexity of structural variation in tumor genomes. Thus, it is important to continue to explore alternative methods for large-scale variant detection in tumor samples. Our method approaches this issue by using multiple references to model potential breakpoints, decreasing the search space in which alignment algorithms function, and overcoming the problem of ‘best’ alignment mapping by allowing all alignments for each read and evaluating each region individually. This is an important step in tumor analysis due to the presence of clinically significant sub-clonal populations with complex chromosomal rearrangements.

## AVAILABILITY

*De novo reference* generation is implemented in Java using the Hadoop MapReduce v1.2.1 framework and HBase 0.94. This can be run on a standard desktop machine (without the benefit of parallel computation) with the standalone installation of Hadoop. It may also be run on a cluster which uses Hadoop locally or through Amazon EC2. A compiled version is available at http://sourceforge.net/projects/insilicogenome/files/releases/HBase-Genomes-1.2.jar the corresponding HBase database is available at http://sourceforge.net/projects/insilicogenome/files/Databases/GRCh37.tgz

Analysis of the resulting BAM files is performed in R v3.0.1, available at http://sourceforge.net/projects/insilicogenome/files/releases/denovo_analysis-1.2.tgz

All source code is available on Github (see README files in each module) at https://github.com/skillcoyne/IGCSA

## Supplementary Material

SUPPLEMENTARY DATA
