# Supplementary material for: Identification of large-scale genomic variation in cancer genomes using in silico reference models
Source: Nucleic Acids Res. 2015 Aug 11;44(1):e5. doi: 10.1093/nar/gkv828 (PMC4705683; doi:10.1093/nar/gkv828)
Supplement: SUPPLEMENTARY DATA [file supp_44_1_e5__index.html]

Identification of large-scale genomic variation in cancer genomes using in silico reference models — Identification of large-scale genomic variation in cancer genomes using in silico reference models — SUPPLEMENTARY DATA 

# Identification of large-scale genomic variation in cancer genomes using *in silico* reference models

## SUPPLEMENTARY DATA

- SUPPLEMENTARY DATA
- SUPPLEMENTARY DATA
- SUPPLEMENTARY DATA
