# Supplementary material for: Identification of large-scale genomic variation in cancer genomes using in silico reference models
Source: Nucleic Acids Res. 2015 Aug 11;44(1):e5. doi: 10.1093/nar/gkv828 (PMC4705683; doi:10.1093/nar/gkv828)
Supplement: SUPPLEMENTARY DATA [file supp_gkv828_nar-01403-met-n-2015-File009.docx]

**Differential Evolution Algorithm for Region Selection**

The optimization algorithm works on individual solutions that are karyotype representations. The information to both create and assess the individual candidate solutions is provided by the breakpoint frequencies obtained from the public karyotypes. However, it is also important that the solution set is not overly biased to only the most probable variants as would be likely if a simple search was performed based on frequencies alone. This means each individual karyotype is assessed in two parts:

***How similar is this karyotype to all others in the populations?*** The similarity of any single individual to any other is assessed through a weighted graph. Each individual is connected to all other individual, the edge between each pair is weighted by the Normalized Compression Distance (1, 2) or NCD, between each pair of individuals (see Equation 1). The NCD is calculated based on the compressed string representation of the breakpoints (the strings are preformatted by ordering and removing extraneous characters “9p24,4q28”),

| Normalized Compression Distance  $\boldsymbol{NCD=}\frac{\boldsymbol{C}_{\boldsymbol{xy}}\boldsymbol{-min}\left\{ \boldsymbol{C}_{\boldsymbol{x}} \right.\boldsymbol{,}\left. \boldsymbol{C}_{\boldsymbol{y}} \right\}}{\boldsymbol{max}\left\{ \boldsymbol{C}_{\boldsymbol{x}} \right.\boldsymbol{,}\left. \boldsymbol{C}_{\boldsymbol{y}} \right\}}$ |
| --- |

where $C_{x}$ and $C_{y}$ are the compression scores of the individuals and $C_{xy}$ is the compression of both together. The closer to zero the NCD is, the more similar the two individuals are.

***How probable is the individual karyotype?*** Fitness for an individual karyotype (see Equation 2) is assessed based on the probabilities calculated from the karyotype data for each breakpoint in the individual solution, and the edge weights representing the similarity between the single solution and all others in the population. It is then adjusted for the number of represented breakpoints per chromosomes, which penalizes individuals that have a nearly 1:1 ratio of chromosomes and breakpoints (the populations rapidly resolves to every individual having maximum breakpoints without this).

| Karyotype Fitness Function  $F_{k}=\sum\left( P\left( {bp}_{1\vert n} \right), \left( \sum_{k=1}^{n} NCD \right)e^{-1}, \frac{{chr}_{count}}{{bp}_{count}} \right)$ | , |
| --- | --- |

where $P\left( {bp}_{1|n} \right)$ is the summed probability of all breakpoints within the individual (1 to *n*), the *NCD* is summed pairwise (using the edge weights in a graph) between the individual and all others in the population, and the ratio of the total count of chromosomes to breakpoints.

These functions are used in the optimization of the population being searched, in the DE this is in the Selection step where the population is evaluated and filtered for individuals to be used in the next iteration. In the Selection step there are two tests applied to the individual solutions. Each individual is checked for its similarity (NCD) against all other individuals. If one is found to have a NCD below a minimum threshold (0.2 was selected as the threshold that maximized diversity) the pair is run through a Tournament-like selection. Essentially, one of the two is randomly selected to stay in the population and the other is removed in the following generation. The second step is to remove individuals with either “perfect” fitness (e.g. no breakpoints at all) or a score that is too above a maximum threshold (e.g. meaning that a very high number of breakpoints or chromosomes have been represented in this individual).

The algorithm is terminated when the population reaches either a maximum number of unrepresented breakpoints across all possible bands (e.g. 3-10), or 1000 generations have been run. With a population of 200 the algorithm typically reaches an optimal point with regards to breakpoint representation within 400 generations.

**References**

1. Bennett,C.H., Gács,P., Li,M., Vitányi,P.M.B. and Zurek,W.H. (1998) Information distance. *Inf. Theory, IEEE Trans.*, **44**, 1407–1423.

2. Cilibrasi,R. and Vitányi,P.M.B. (2005) Clustering by Compression. *IEEE Trans. Inf. Theory*, **51**, 1523–1545.
